# Impact of Early Nonfatal Complications After Transcatheter Aortic Valve Implantation on 1-Year Mortality and Quality of Life: Insights From the LANDMARK Trial

**DOI:** 10.1016/j.shj.2026.101101

**Published:** 2026-07-24

**Authors:** Akihiro Tobe, Yoshinobu Onuma, Niels van Royen, Ignacio J. Amat-Santos, Martin Hudec, Matjaz Bunc, Ben J.L. van den Branden, Peep Laanmets, Daniel Unic, Bela Merkely, Renicus S. Hermanides, Vlasis Ninios, Marcin Protasiewicz, Benno J.W.M. Rensing, Pedro L. Martin, Fausto Feres, Manuel de Sousa Almeida, Eric Van Belle, Axel Linke, Alfonso Ielasi, Matteo Montorfano, Mark Webster, Konstantinos Toutouzas, Emmanuel Teiger, Francesco Bedogni, Michiel Voskuil, Manuel Pan, Oskar Angerås, Won-Keun Kim, Jürgen Rothe, Mohamed Abdel-Wahab, Ivica Kristić, Vicente Peral, Scot Garg, Ashokkumar Thakkar, Udita Chandra, Pieter C. Smits, Marie-Claude Morice, Andreas Baumbach, Patick W. Serruys

**Affiliations:** a CORRIB Research Centre for Advanced Imaging and Core Laboratory, University of Galway, Galway, Ireland; b Department of Cardiology, Radboud University Medical Center, Nijmegen, the Netherlands; c Centro de Investigación Biomédica en Red - Enfermedades Cardiovasculares (CIBERCV), Instituto de Salud Carlos III, Madrid, Spain; d Department of Cardiology, Hospital Clínico Universitario de Valladolid, Valladolid, Spain; e Department of Acute Cardiology, Middle-Slovak Institute of Cardiovascular Diseases, Banska Bystrica, Slovakia; f Department of Cardiology, University Medical Centre Ljubljana, Ljubljana, Slovenia; g Department of Cardiology, Amphia Hospital, Breda, the Netherlands; h Department of Invasive Cardiology, North Estonia Medical Centre, Tallinn, Estonia; i Department of Cardiac and Transplant Surgery, University Hospital Dubrava, Zagreb, Croatia; j Heart and Vascular Center, Semmelweis University, Budapest, Hungary; k Department of Cardiology, Isala Hospital, Zwolle, the Netherlands; l Department of Cardiology, European Interbalkan Medical Center, Thessaloniki, Greece; m Department of Cardiology, Institute of Heart Diseases, Wroclaw Medical University, Wroclaw, Poland; n Faculty of Medicine, Department of Non-Procedural Clinical Sciences, Wroclaw University of Science and Technology, Wroclaw, Poland; o Department of Cardiology, St Antonius Hospital, Nieuwegein, the Netherlands; p Department of Interventional Cardiology, University Hospital of Gran Canaria Dr Negrín, Las Palmas de Gran Canaria, Spain; q Department of Invasive Cardiology, Instituto Dante Pazzanese, São Paulo, Brazil; r CHRC, NOVA Medical School, NOVA University Lisbon, Lisbon, Portugal; s Department of Interventional Cardiology, Lille University Hospital, Lille, France; t Department of Internal Medicine and Cardiology, Heart Center Dresden, University of Technology Dresden, Dresden, Germany; u Cardiologia Ospedaliera, IRCCS Ospedale Galeazzi Sant’Ambrogio, Milan, Italy; v School of Medicine, Vita-Salute San Raffaele University, Milan, Italy; w Interventional Cardiology Unit, IRCCS San Raffaele Scientific Institute, Milan, Italy; x Department of Cardiology, Auckland City Hospital, Auckland, New Zealand; y Department of Cardiology, Hippokration Hospital, Athens, Greece; z Department of Cardiology, Henri Mondor University Hospital, Assistance Publique–Hôpitaux de Paris (AP-HP), Créteil, France; aa Department of Clinical Cardiology, IRCCS Policlinico San Donato, San Donato Milanese, Italy; ab Department of Cardiology, University Medical Center Utrecht, Utrecht, the Netherlands; ac Department of Cardiology, University Hospital Reina Sofía, University of Córdoba, IMIBIC, CIBERCV, Córdoba, Spain; ad Department of Thoracic Surgery and Cardiology, Sahlgrenska University Hospital, Gothenburg, Sweden; ae Department of Molecular and Clinical Medicine, Institute of Medicine, Sahlgrenska Academy, University of Gothenburg, Gothenburg, Sweden; af Department of Cardiology & Angiology, University of Giessen and Marburg, Gießen, Germany; ag Department of Cardiology, Kerckhoff Heart Center, Bad Nauheim, Germany; ah Department of Cardiology and Angiology, Campus Bad Krozingen, University Heart Center-University of Freiburg, Bad Krozingen, Germany; ai Faculty of Medicine, University of Freiburg, Freiburg, Germany; aj Department of Structural Heart Disease/Cardiology, Heart Center Leipzig at Leipzig University, Leipzig, Germany; ak Department of Cardiology, University Hospital of Split, Split, Croatia; al Department of Cardiology University Hospital Son Espases, Health Research Institute of the Balearic Islands, Palma de Mallorca, Spain; am Department of Cardiology, Royal Blackburn Hospital, Blackburn, UK; an School of Medicine, University of Central Lancashire, Preston, UK; ao Department of Clinical Research, Meril Life Sciences Pvt. Ltd., Vapi, India; ap Cardiovascular European Research Center (CERC), Massy, France; aq ICPS, Hôpital Privé Jacques Cartier, Massy, France; ar Centre for Cardiovascular Medicine and Devices, William Harvey Research Institute, Queen Mary University of London and Barts Heart Centre, London, UK; as Cleveland Clinic, London, UK

**Keywords:** Complication, Mortality, Quality of life, Randomized controlled trial, SF-12, Transcatheter aortic valve implantation

## Abstract

**Background:**

The impact of early nonfatal complications on subsequent mortality and quality of life (QOL) after contemporary transcatheter aortic valve implantation (TAVI) remains unclear.

**Methods:**

This post-hoc substudy of the LANDMARK trial included patients with severe aortic stenosis randomized to the Myval transcatheter heart valve (THV) series or to contemporary THV (Sapien or Evolut) series. Early nonfatal complications were defined as events occurring within 30 days after TAVI in patients surviving the first 30 days and included stroke, type 3 bleeding, major vascular complications, acute kidney injury stages 2-4, moderate or severe aortic regurgitation, new permanent pacemaker implantation, or reintervention according to the VARC-3 early safety endpoint. The primary endpoint was all-cause mortality from 30 to 365 days. QOL was assessed using the Short Form-12 at baseline, 30 days, and 1 year.

**Results:**

Of the 768 randomized patients, 742 were alive at 30 days. Of these, 177 (23.9%) experienced at least 1 early nonfatal complication, whereas 565 (76.1%) did not. Age (80 ± 5 years vs. 80 ± 6 years, *p* = 0.67) and Society of Thoracic Surgeons score (median 2.6 [interquartile range: 1.6-3.9] vs. 2.6 [1.7-3.9], *p* = 0.76) were similar between groups. Patients with early nonfatal complications had higher mortality between 30 and 365 days (8.0% vs. 3.9%, *p* = 0.03). Early nonfatal complications were independently associated with increased 1-year mortality (hazard ratio = 2.14; 95% CI, 1.07-4.27; *p* = 0.03). One-year QOL did not differ between groups.

**Conclusions:**

In this post-hoc, hypothesis-generating analysis, early nonfatal complications after TAVI were associated with increased 1-year mortality, whereas 1-year QOL was comparable among survivors; findings should be interpreted with caution due to potential survivor bias.

**Trial registration number:**

ClinicalTrials.gov, NCT04275726.

## Highlights

• Early nonfatal complications after transcatheter aortic valve implantation is associated with higher 1-year mortality.
• Quality of life outcomes at 1 year were similar between those with and without complications.
• Efforts should focus on the prevention of complications and their optimal management.

## Graphical abstract

**Figure:**
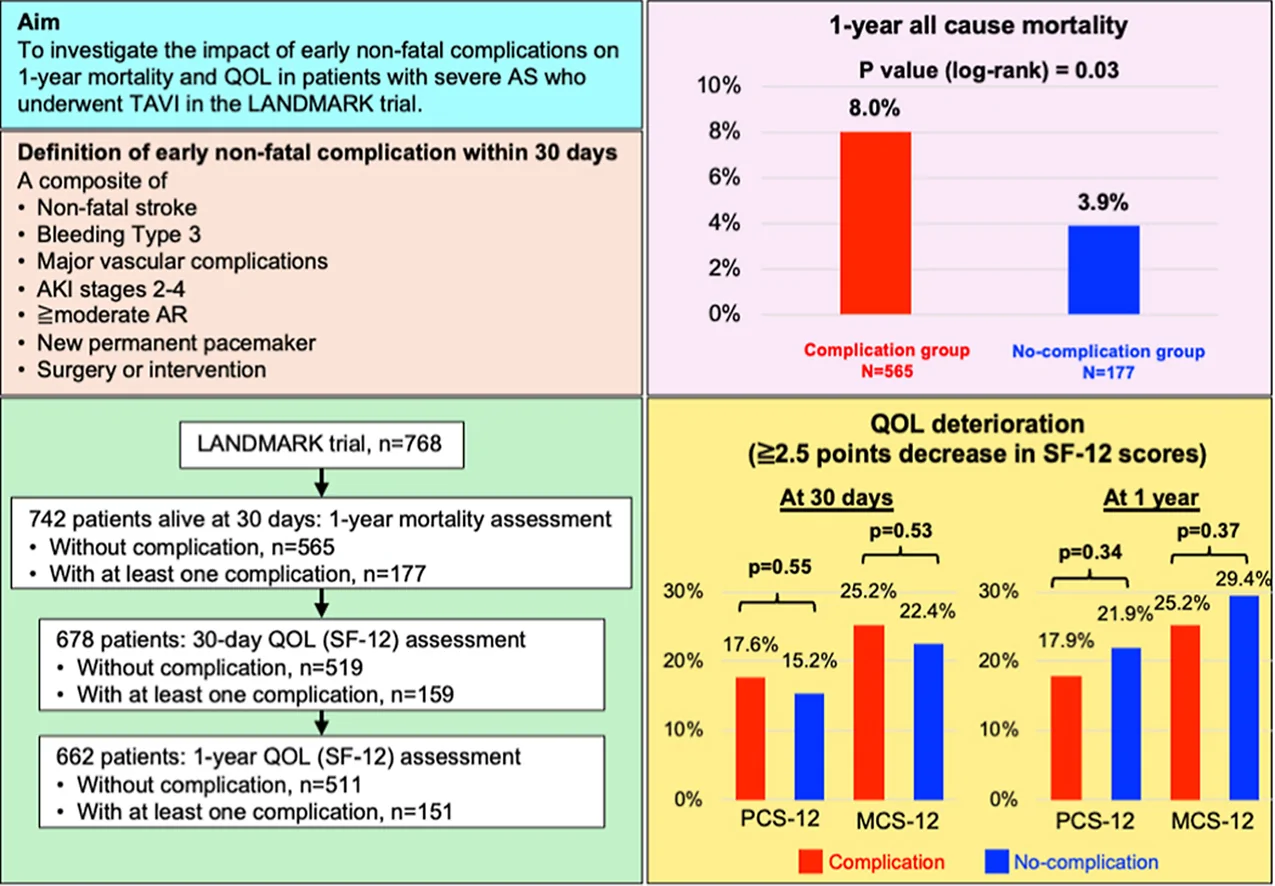

## Introduction^1^

Aortic stenosis (AS) is a potentially life-threatening cardiac condition if left untreated. Moreover, its associated symptoms, such as the classical triad of dyspnea, angina, and syncope, significantly impair quality of life (QOL). Transcatheter aortic valve implantation (TAVI) is an established alternative to surgical aortic valve replacement (SAVR) for treating severe AS.^1^ Its safety and effectiveness in terms of hard clinical endpoints, such as mortality and stroke, are comparable to, or even better than, those of SAVR.^2^^,^^3^ Compared with SAVR, TAVI is less invasive and allows faster postprocedural recovery, resulting in shorter hospital stays and better short-term QOL.^4^

Despite the less invasive nature of TAVI and ongoing technological advancements, a subset of patients still experience periprocedural or early postprocedural complications. Recent real-world data from the Society of Thoracic Surgeons (STS)/American College of Cardiology Transcatheter Valve Therapy registry reported that, among nearly 70,000 patients at low surgical risk, the 30-day incidence of a composite endpoint of death, stroke, major or life-threatening bleeding, acute kidney injury (AKI) stage 3 or dialysis, or moderate or severe paravalvular leak was 6.3%, whereas new permanent pacemaker implantation (PPI) occurred in 8.4% of patients.^5^

As TAVI indications expand to younger and less symptomatic patients, further reductions in procedural complications have become a critical challenge in contemporary TAVI practice. Many studies have shown that complications are associated with increased mortality.^6^, ^7^, ^8^, ^9^, ^10^, ^11^, ^12^ However, only a limited number of studies have evaluated the impact of early nonfatal complications on both subsequent mortality and patient-reported QOL.^13^, ^14^, ^15^

Recently, we reported the 1-year results of the LANDMARK trial, which showed comparable clinical and QOL outcomes in patients with symptomatic severe native AS randomized to the TAVI with the Myval transcatheter heart valve (THV) series or the contemporary standard Sapien and Evolut THV series.^16^ In the present study, we investigated the impact of early nonfatal complications after TAVI on subsequent mortality and QOL at 1 year in the LANDMARK trial.

## Methods

### Study Design and Participants of the LANDMARK Trial

This was a post-hoc, hypothesis-generating substudy of the LANDMARK trial. The design and main results of the LANDMARK trial have been previously published.^16^, ^17^, ^18^, ^19^, ^20^ In brief, the LANDMARK trial is a prospective, multicenter, multinational, open-label, noninferiority trial conducted across 31 sites in 16 countries (Germany, France, Sweden, the Netherlands, Italy, Spain, Portugal, Greece, Hungary, Poland, Slovakia, Slovenia, Croatia, Estonia, New Zealand, and Brazil). A total of 768 participants were enrolled and randomly assigned in a 1:1 ratio to either the Myval arm (n = 384) or the contemporary THV arm (Sapien or Evolut series, n = 384). The Myval THV series comprised the Myval THV and Myval Octacor with sizes 20 mm, 21.5 mm, 23 mm, 24.5 mm, 26 mm, 27.5 mm, and 29 mm. The Sapien THV series comprised the Sapien 3 and Sapien 3 Ultra with sizes 20 mm, 23 mm, 26 mm, and 29 mm. The Evolut THV series comprised the Evolut R, Evolut PRO, Evolut PRO+, and Evolut FX with sizes 23 mm, 26 mm, 29 mm, and 34 mm.

The study was approved by the ethics committees of all participating centers and conducted in accordance with Good Clinical Practice guidelines and the Declaration of Helsinki. Eligible participants were adults aged ≥18 years with symptomatic severe native AS deemed suitable for transfemoral TAVI using all 3 study devices. A prescreening committee assessed the appropriateness of TAVI, but the final decision to enroll was made by the local heart team. Clinical outcomes were adjudicated by an independent clinical events committee, blinded to treatment allocation, according to the Valve Academic Research Consortium-3 (VARC-3) criteria.^21^ Transthoracic echocardiography and electrocardiograms at preprocedural baseline, discharge, 30 days, and 1 year were assessed by independent core laboratories (transthoracic echocardiography: CORRIB core lab, Galway, Ireland; electrocardiogram: CERC, Massay, France).

The trial demonstrated that the Myval THV series was noninferior to the contemporary THVs (Sapien and Evolut series) for the 30-day early safety endpoint, and showed comparable performance for the 1-year clinical, hemodynamic, and QOL endpoints.^16^^,^^17^^,^^22^, ^23^, ^24^

### Early Nonfatal Complications

Early nonfatal complications included the following events occurring within 30 days after TAVI in patients who remained alive at 30 days: nonfatal stroke, Type 3 bleeding, major vascular complications, AKI stages 2-4, moderate or severe prosthetic valvular regurgitation (PVR), new PPI, or surgery or intervention related to the device or to a major vascular, access-related, or cardiac structural complication as defined by the VARC-3 early safety endpoint.^21^ All events were adjudicated by the clinical events committee, and PVR was assessed by the echo core lab.

### Outcomes

All patients alive at 30 days after TAVI were included in the present analysis. All-cause mortality was assessed from 30 to 365 days after the index TAVI. In addition, the incidence of stroke, procedure- or valve-related hospitalization, and the clinical efficacy composite endpoint (defined as all-cause death, all stroke, or procedure- or valve-related hospitalization) were also assessed during the same period (30 to 365 days after TAVI).

QOL was assessed at preprocedural baseline, 30 days, and 1 year using the Short Form-12 (SF-12) questionnaire. The SF-12 is a generic health-related QOL assessment tool that is widely used in the cardiovascular field.^25^ It is a shortened version of the original Short Form-36, consisting of 12 items covering the 8 health domains: physical functioning, social functioning, role limitations (physical problems), role limitations (emotional problems), mental health, energy/vitality, bodily pain, and general health.^26^ These 8 domains are aggregated into summary measures: the physical component summary (PCS) and the mental component summary (MCS) scores, with higher scores indicating better health-related QOL. The SF-12 can be completed in approximately 1 minute. In the general population of the United States, the mean PCS and MCS scores are both 50 with an SD of 10. Among individuals aged ≥75 years, the mean PCS and MCS scores are 39 (SD 11) and 50 (SD 11), respectively.^27^

A decrease of ≥2.5 points from baseline was defined as QOL deterioration, based on previously reported minimally clinically important differences in the SF-12 score.^21^^,^^28^

Mortality was assessed in patients who underwent TAVI, were alive at 30 days, and had not withdrawn consent at 30 days, regardless of the availability of SF-12 scores. Changes in SF-12 scores at 30 days and 1 year were assessed in patients with available baseline and corresponding 30-day or 1-year SF-12 scores, respectively. Multiple imputations were performed for patients with missing 1-year SF-12 data, who were alive at 1 year and had not withdrawn consent.

### Statistical Analysis

For baseline characteristics, continuous variables are presented as mean ± SD or median [interquartile range], as appropriate. *p*-values were calculated using a 2-sample t-test comparing patients with and without early nonfatal complications. Categorical variables are presented as counts and percentages and compared using Pearson’s χ^2^ test or Fisher exact test, as appropriate. Mortality and other clinical outcomes were assessed from 30 to 365 days after TAVI using Kaplan–Meier estimates. Log-rank tests were used to compare survival between patients without early nonfatal complications and those with any early complication, as well as between patients without complications and those with each individual complication component. Changes in SF-12 scores at 30 days (ΔSF-12 at 30 days) were calculated as the difference between the 30-day and baseline SF-12 scores (30-day minus baseline SF-12 scores) and are presented as mean ± SD. Changes in SF-12 scores at 1 year (ΔSF-12 at 1 year) were calculated as the difference between 1-year and baseline SF-12 scores (1-year minus baseline SF-12 scores) and are presented as mean ± SD. These changes were calculated only for patients with SF-12 assessments available at both baseline and the corresponding follow-up point. Between-group comparisons used the two-sample t-test. The proportion of patients with clinically meaningful deterioration in SF-12 scores (decrease of ≥2.5 points from baseline) was compared between groups using the chi-square test or Fisher's exact test, as appropriate. Univariable and multivariable cox regression analyses were performed to identify predictors of 1-year all-cause mortality. Variables which were statistically significant in the univariable analysis were included in the multivariable model.

Missing SF-12 data at 1 year among patients who were alive and had not withdrawn consent were imputed using multiple imputation by the chained equation method.^16^ This method assumes that data are missing at random and iteratively imputes missing values based on observed data. The following parameters were used for multiple imputation for missing SF-12: age, gender, body mass index, STS Predicted Risk of Mortality (PROM) (STS-PROM) score, hypertension, chronic obstructive pulmonary disease, diabetes, previous stroke, coronary artery disease, peripheral vascular disease, previous myocardial infarction, previous percutaneous coronary intervention, previous coronary artery bypass grafting, bicuspid valve, mitral insufficiency, aortic annulus area, mean pressure gradient, stroke volume, left ventricular ejection fraction, PCS and MCS at preprocedural baseline and 30 days, 6 minute-walk test distance at preprocedural baseline and 30 days, and New York Heart Association functional class at preprocedural baseline. Sensitivity analyses were also performed without imputing missing SF-12 scores.

Statistical analyses were performed using R software (version 4.3.3; R Foundation for Statistical Computing, Vienna, Austria).

### Data Availability

The LANDMARK trial plans to continue follow-up for up to 10 years. Patient-level data collected for this study will not be made publicly available but can be shared upon request after the final long-term follow-up is published.

## Results

**Figure 1:**
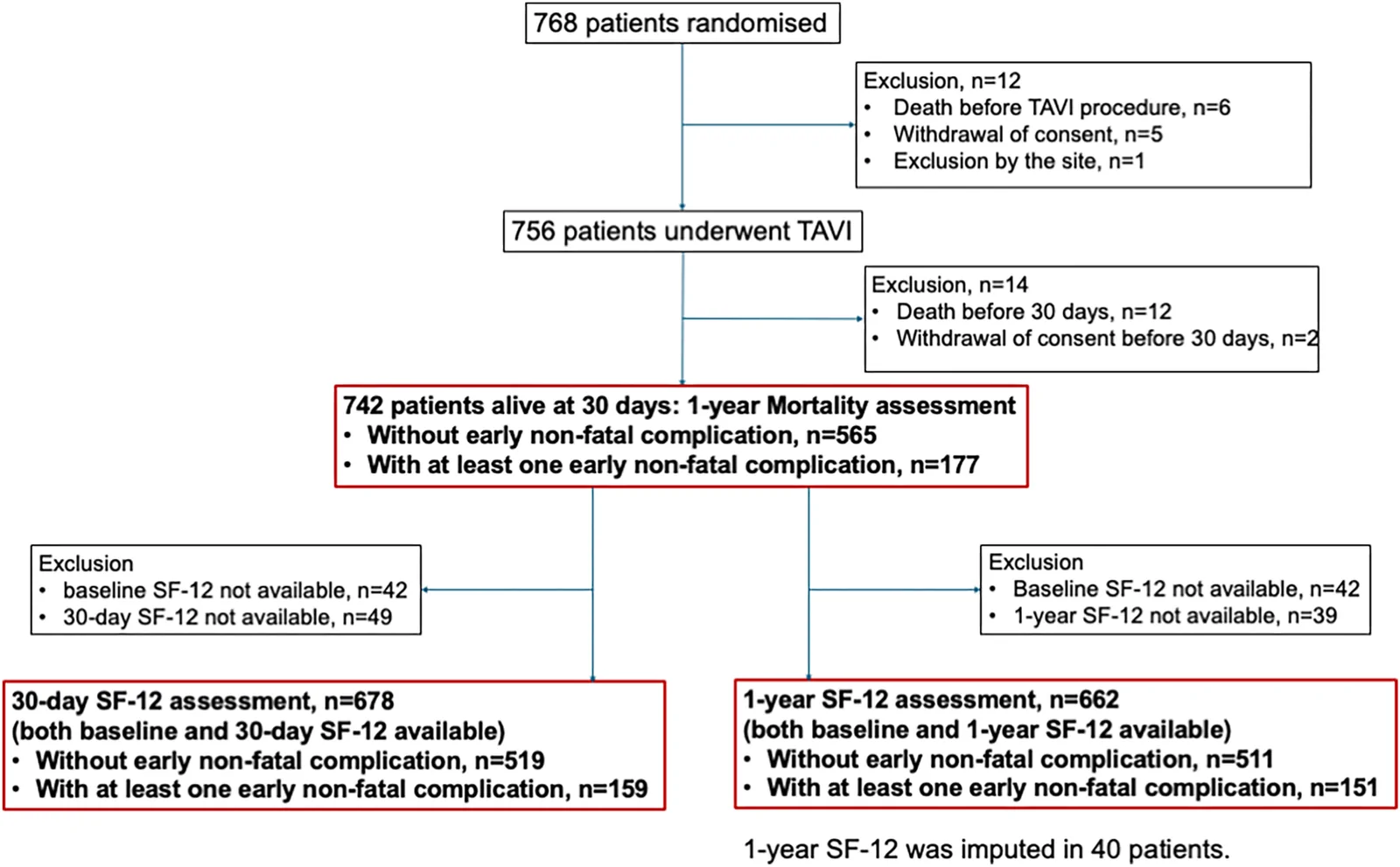
**Patient flowchart.** One-year mortality was assessed in 742 patients who were alive and had not withdrawn their consent at 30 days. QOL was assessed using SF-12. Thirty-day QOL was assessed among patients in whom both preprocedural baseline and 30-day SF-12 were available. One-year QOL was assessed in 662 patients with both preprocedural baseline and 1-year SF-12 available. Multiple imputation was performed for 40 patients with missing SF-12 at 1 year who were alive and had not withdrawn their consent. Sensitivity analysis was also performed excluding these 40 patients with imputation. Abbreviations: QOL, quality of life; SF-12, Short Form-12; TAVI, transcatheter aortic valve implantation.

Of the 768 randomized patients, 756 underwent TAVI, and 742 were alive at 30 days and had not withdrawn consent; these patients comprised the cohort for the 1-year mortality analysis. Six patients had died before TAVI, and 12 died within 30 days after TAVI. All 18 deaths were cardiovascular deaths, of which 8 were procedure-related (Figure 1). Among the 742 patients in the analysis cohort, 177 (23.9%) experienced at least 1 early nonfatal complication (complication group), whereas 565 (76.1%) did not (no-complication group). Early nonfatal complications included (not mutually exclusive) nonfatal stroke (n = 18; disabling, n = 7; nondisabling, n = 11), type 3 bleeding (n = 10), major vascular complications (n = 12), AKI stages 2–4 (n = 8), moderate PVR (n = 27), new PPI (n = 120), and surgery or intervention (n = 14). The occurrence of early nonfatal complications according to valve type is presented in Supplementary Table 1.

Baseline and procedural characteristics of the 742 patients alive at 30 days are shown in Table 1. Patients with early nonfatal complications had a significantly higher prevalence of atrial fibrillation or flutter (31% vs. 23%, *p* = 0.03) and baseline right bundle branch block (19% vs. 8%, *p* < 0.01). The proportion of male patients tended to be higher (58% vs. 50%, *p* = 0.07) and left ventricular ejection fraction tended to be lower (57% ± 11% vs. 58% ± 10%, *p* = 0.06) in the complication group. The mean age (80 ± 5 years vs. 80 ± 6 years, *p* = 0.67), median [interquartile range] STS-PROM score (2.6 [1.6–3.9] vs. 2.6 [1.7–3.9], *p* = 0.76), and THV type (Myval 46% vs. 49%, Sapien 24% vs. 27%, and Evolut 29% vs. 24%, *p* = 0.30) did not differ significantly between groups.

**Table 1:** Baseline and procedural characteristics in patients who were alive at 30 d

Characteristics	With early complications (n = 177)	Without early complications (n = 565)	*p* value
Age (y)	80.3 ± 5.3	80.1 ± 5.7	0.67
Male sex	103 (58.2%)	282 (49.9%)	0.07
Body mass index (kg/m^2^)	28.19 ± 5.4	28.1 ± 4.7	0.84
STS score (%)	2.55 [1.61, 3.93]	2.58 [1.71, 3.91]	0.76
Low risk (<4%)	134 (75.7%)	430 (76.1%)	0.99
Intermediate risk (4%-8%)	34 (19.2%)	114 (20.2%)	0.86
High risk (>8%)	9 (5.1%)	21 (3.7%)	0.56
Diabetes	50 (28.3%)	166 (29.4%)	0.85
Dyslipidemia	23 (13.0%)	54 (9.6%)	0.24
Hypertension	113 (63.8%)	376 (66.6%)	0.57
eGFR <60 ml/min	76/168 (45.2%)	259/534 (48.5%)	0.52
eGFR <30 ml/min	18/168 (10.7%)	83/534 (15.5%)	0.15
Chronic obstructive pulmonary disease	15 (8.5%)	64 (11.3%)	0.35
History of atrial fibrillation or flutter	55 (31.1%)	129 (22.8%)	0.03
Previous stroke	4 (2.3%)	15 (2.7%)	0.10
Previous MI	12 (6.8%)	35 (6.2%)	0.92
Previous CABG	9 (5.1%)	24 (4.3%)	0.79
Previous PCI	16 (9.0%)	37 (6.6%)	0.34
NYHA III/IV	90 (50.9%)	298 (52.7%)	0.72
6MWT (m)	282 ± 115	275 ± 107	0.49
Preprocedural electrocardiogram	n = 177	n = 561	
LBBB	13 (7.3%)	31 (5.5%)	0.46
RBBB	34 (19.2%)	42 (7.5%)	<0.01
Atrial fibrillation	43 (24.3%)	99 (17.7%)	0.06
Preprocedural echocardiogram			
LVEF (%)	56.6 ± 10.9	58.4 ± 10.0	0.06
Mean pressure gradient (mmHg)	39.0 ± 15.3	39.5 ± 13.3	0.64
Effective orifice area (cm^2^)	0.75 ± 0.23	0.72 ± 0.22	0.17
Preprocedural CT			
Small annulus (≤430 mm^2^)	50 (28.3%)	185 (32.7%)	0.30
Bicuspid valve	14 (7.9%)	38 (6.7%)	0.71
Aortic valve calcification (mm^3^)	1091 ± 769	986 ± 670	0.08
None	2 (1.1%)	4 (0.7%)	0.63
Mild	29 (16.4%)	96 (17.0%)	0.94
Moderate	56 (31.6%)	217 (38.5%)	0.12
Severe	90 (50.9%)	247 (43.8%)	0.12
Procedural characteristics			
Valve type∗			
Myval	81 (45.8%)	278 (49.2%)	0.48
Sapien	43 (24.3%)	150 (26.5%)	0.62
Evolut	52 (29.4%)	135 (23.9%)	0.17
Myval and Sapien	0 (0%)	1 (0.2%)	1.00
Evolut and Evolut	0 (0%)	1 (0.2%)	1.00
Portico	1 (0.6%)	0 (0%)	0.24
Predilatation	81 (45.8%)	221 (39.1%)	0.14
Postdilatation	34 (19.2%)	81 (14.3%)	0.15
Cerebral protection device	18/171 (10.5%)	62/534 (11.6%)	0.80

Data are presented as mean ± SD, median [Q1, Q3], or n (%), as appropriate.

Abbreviations: 6MWT, 6-minute walk test; CABG, coronary artery bypass grafting; CT, computed tomography; eGFR, estimated glomerular filtration rate; LBBB, left bundle branch block; LVEF, left ventricle ejection fraction; MI, myocardial infarction; NYHA, New York Heart Association; PCI, percutaneous coronary intervention; RBBB, right bundle branch block; STS, Society of Thoracic Surgeons.

∗ Analysis was restricted to Myval/Sapien/Evolut valve categories because of extremely small numbers in other categories.

### Mortality and Other Clinical Outcomes at 1 Year

**Figure 2:**
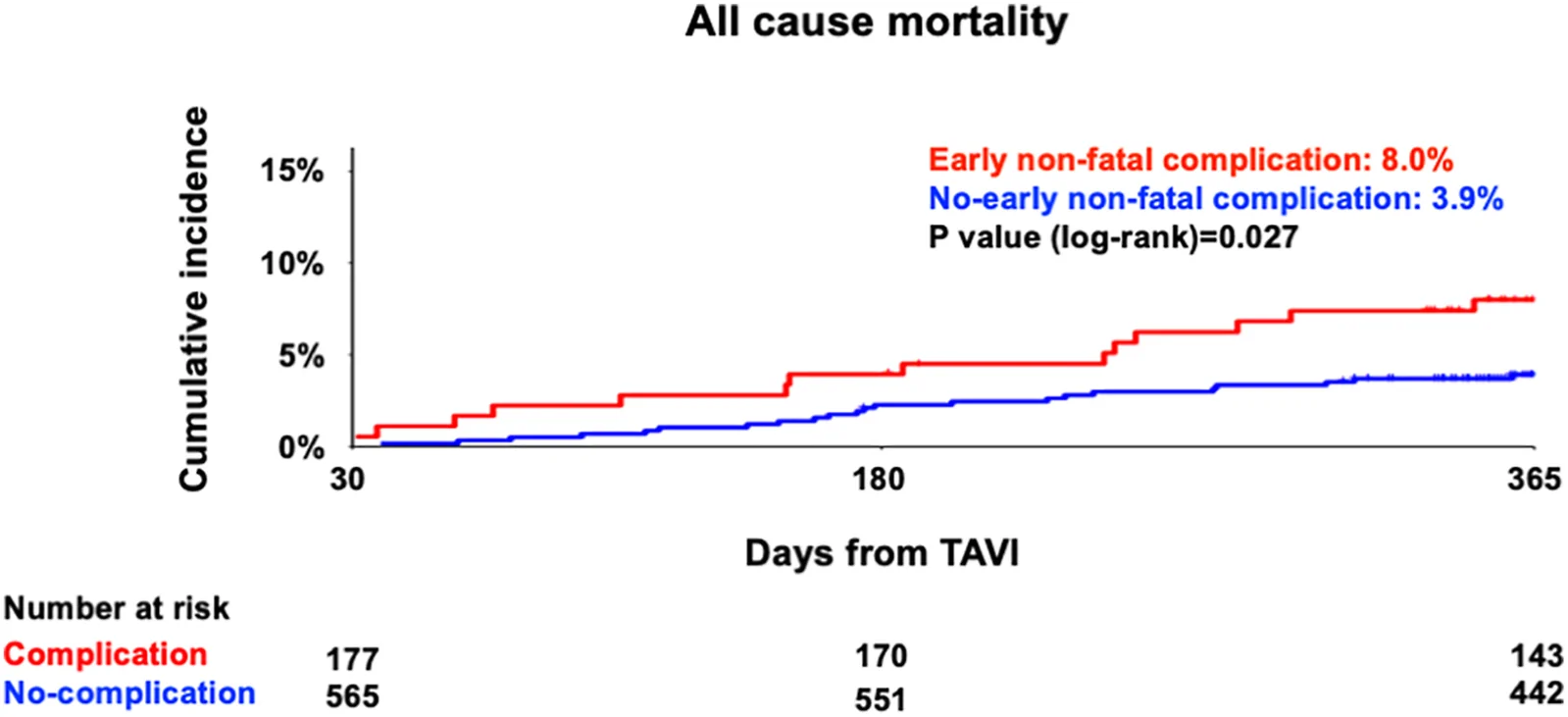
**Cumulative incidence of all-cause mortality from 30 days to 1 year.** Abbreviation: TAVI, transcatheter aortic valve implantation.

The mortality rate between 30 and 365 days after the index TAVI was significantly higher in the complication group than in the no-complication group (8.0%, n = 14 vs. 3.9%, n = 22, *p* = 0.03; Table 2 and Figure 2). In the complication group, there were no deaths among patients with nonfatal stroke or type 3 bleeding; however, patients with major vascular complications, AKI stages 2-4, and moderate PVR had significantly higher 1-year mortality than the no-complication group (major vascular complications: n = 2 [16.7%], *p* = 0.03; AKI stage 2-4, n = 3 [37.5%], *p* < 0.01; moderate PVR, n = 5 [18.5%], *p* < 0.01). There was an overlap of 4 patients between the type 3 bleeding and major vascular complication groups. The 2 patients who died in the major vascular complication group had major vascular complications alone, without concomitant type 3 bleeding. No significant between-group differences in 1-year mortality were observed among patients with either new PPI (n = 8 [6.7%], *p* = 0.16) or surgery/intervention (n = 1 [7.1%], *p* = 0.50; Table 2). The forest plot of hazard ratios (HRs) for each complication is shown in Supplementary Figure 1. All-cause mortality stratified by the number of early nonfatal complications is presented in Supplementary Figure 2.

**Table 2:** Mortality from 30 to 365 d after TAVI

	All-cause mortality n (%)	*p* value
Patients without complications within 30 d, n = 565	22 (3.9%)	-
Patients with any complications within 30 d∗, n = 177	14 (8.0%)	0.03
Nonfatal stroke∗, n = 18 (2.4%)	0 (0.0%)	0.38
Disabling stroke∗, n = 7 (0.9%)	0 (0.0%)	0.57
Nondisabling stroke∗, n = 11 (1.5%)	0 (0.0%)	0.49
Bleeding type 3∗, n = 10 (1.3%)	0 (0.0%)	0.51
Major vascular complications∗, n = 12 (1.6%)	2 (16.7%)	0.03
AKI stage 2-4∗, n = 8 (1.1%)	3 (37.5%)	<0.01
≧moderate PVR∗, n = 27 (3.6%)	5 (18.5%)	<0.01
New permanent pacemaker∗, n = 120 (16.2%)	8 (6.7%)	0.16
Surgery or intervention related to the device, n = 14 (1.9%)	1 (7.1%)	0.50

All-cause mortality from 30 days to 365 days after TAVI in patients who were alive at 30 days.

*p* values were calculated using log-rank test between patients without early non-fatal complications and those with complications.

Abbreviations: AKI, acute kidney injury; PVR, prosthetic valvular regurgitation; TAVI, transcatheter aortic valve implantation.

∗ Not mutually exclusive.

The complication group also had significantly higher rates of procedure- or valve-related hospitalizations (n = 10 [5.8%] vs. n = 6 [1.1%], *p* < 0.01) and of the clinical efficacy composite endpoint at 1 year (n = 22 [12.5%] vs. n = 33 [5.9%], *p* < 0.01, Supplementary Table 1).

In the multivariable Cox regression analysis, early nonfatal complications were independently associated with increased 1-year mortality (HR = 2.14; 95% CI, 1.07 - 4.27; *p* = 0.03, Table 3).

**Table 3:** Cox regression analysis for predicting 1-y all-cause mortality (from 30 to 365 d)

Variables	Univariable analysis		Multivariable analysis	
	HR (95% CI)	*p* value	HR (95% CI)	*p* value
Presence of any of early nonfatal complications	2.08 (1.06, 4.07)	0.03	2.14 (1.07, 4.27)	0.03
Age	1.04 (0.98, 1.11)	0.18		
Male	2.16 (1.06, 4.39)	0.03	2.81 (1.31, 6.04)	0.01
Body mass index	1.01 (0.94, 1.08)	0.80		
STS score, continuous value	1.10 (1.02, 1.20)	0.02	1.14 (1.03, 1.26)	0.01
STS categorical value				
Low risk (<4%)	Reference	-		
Intermediate risk (4%-8%)	1.61 (0.77, 3.38)	0.20		
High risk (>8%)	1.59 (0.38, 6.75)	0.53		
Diabetes	1.98 (1.03, 3.82)	0.04	1.58 (0.78, 3.22)	0.20
Hypertension	1.17 (0.58, 2.38)	0.66		
Dyslipidemia	0.24 (0.03, 1.77)	0.16		
eGFR <60 ml/min	1.24 (0.63, 2.43)	0.54		
eGFR <30 ml/min	2.17 (1.01, 4.66)	0.046	1.83 (0.82, 4.11)	0.14
Chronic obstructive pulmonary disease	1.68 (0.70, 4.04)	0.25		
History of atrial fibrillation or flutter	2.24 (1.15, 4.34)	0.02	1.92 (0.96, 3.84)	0.07
Previous stroke	1.09 (0.15, 7.98)	0.93		
Permanent pacemaker	3.26 (1.15, 9.21)	0.03		
Previous MI	0.41 (0.06, 3.02)	0.38		
Previous CABG	0.60 (0.08, 4.39)	0.62		
Previous PCI	1.19 (0.37, 3.88)	0.77		
Preprocedural electrocardiogram				
RBBB	1.76 (0.73, 4.23)	0.21		
LBBB	0.94 (0.23, 3.92)	0.94		
Preprocedural echocardiogram				
LVEF	0.97 (0.94, 1.00)	0.06		
Mean pressure gradient	0.98 (0.95, 1.00)	0.07		
Preprocedural CT				
Small annulus (≤430 mm^2^)	0.99 (0.97, 1.02)	0.59		
Bicuspid valve	1.20 (0.37, 3.92)	0.76		
Valve calcification, mm^3^, continuous value	1.00 (1.00, 1.00)	0.65		
Procedural characteristics				
Valve type				
Myval	Reference	-		
Sapien	0.82 (0.36, 1.88)	0.63		
Evolut	1.08 (0.50, 2.34)	0.85		
General anesthesia	0.99 (0.44, 2.27)	0.99		
Predilatation	0.72 (0.36, 1.44)	0.35		
Postdilatation	1.11 (0.46, 2.67)	0.81		
Cerebral protection device	0.27 (0.04, 1.97)	0.20		

Abbreviations: CABG, coronary artery bypass grafting; CT, computed tomography; eGFR, estimated glomerular filtration rate; LBBB, left bundle branch block; LVEF, left ventricle ejection fraction; MI, myocardial infarction; PCI, percutaneous coronary intervention; RBBB, right bundle branch block; STS, Society of Thoracic Surgeons; HR, hazard ratio.

### QOL at 30 Days

**Figure 3:**
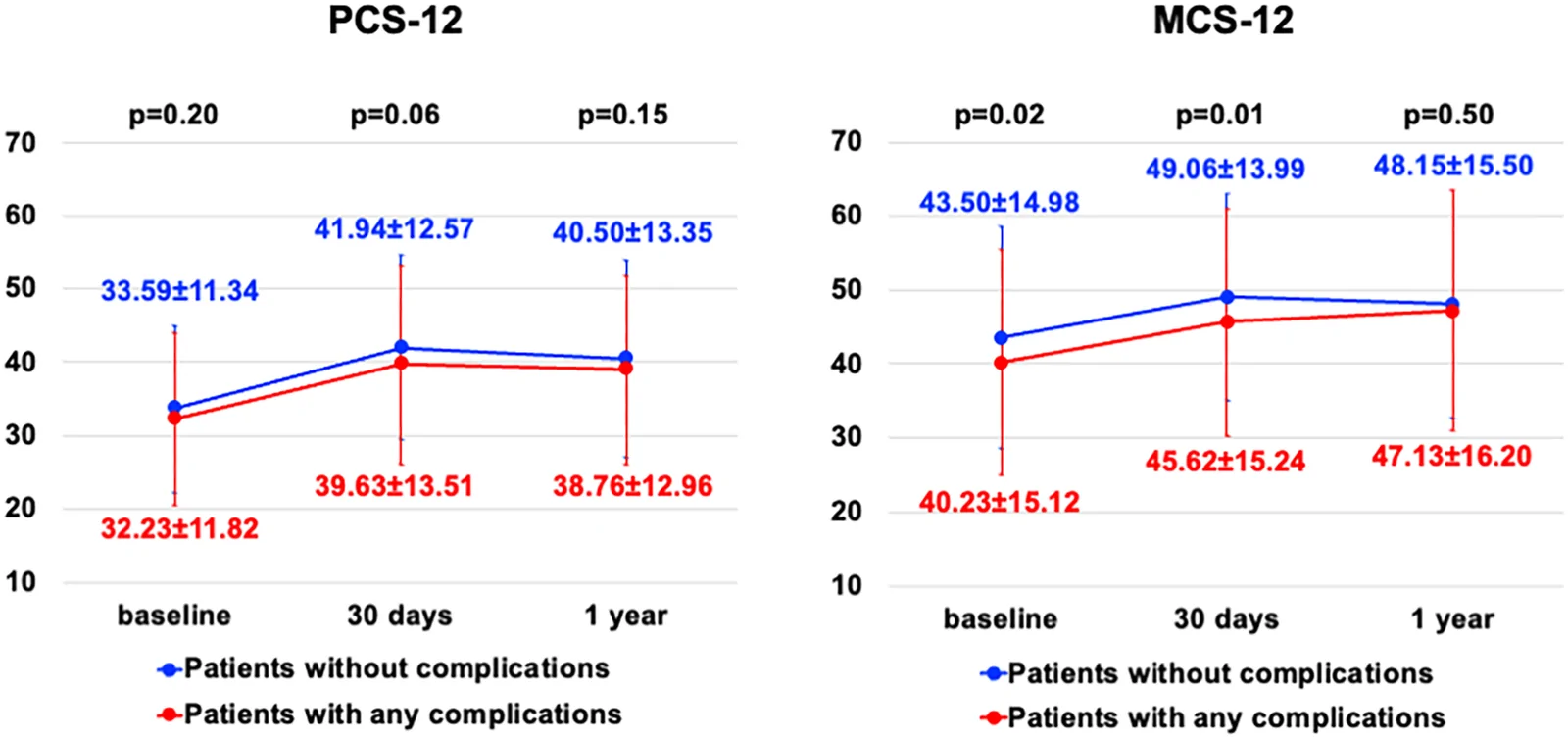
**SF-12 scores at preprocedural baseline, 30 days,and 1 year.***p* values are for the comparison between patients with and without early nonfatal complications at each time point. Abbreviations: MCS, mental component summary; PCS, physical component summary; SF-12, Short Form-12.

Both baseline and 30-day SF-12 scores were available for 678 patients (Figure 1). The absolute values of SF-12 scores at baseline and 30 days are presented in Table 4. The baseline PCS-12 score did not differ significantly between the complication and no-complication groups (32.23 ± 11.82 vs. 33.59 ± 11.34, *p* = 0.20), whereas the baseline MCS-12 was significantly lower in the complication group (40.23 ± 15.12 vs. 43.50 ± 14.98, *p* = 0.02, Figure 3). At 30 days, the MCS-12 score remained significantly lower in the complication group (45.62 ± 15.24 vs. 49.06 ± 13.99, *p* = 0.01). There was no significant difference in the incidence of QOL deterioration at 30 days, defined as ≥2.5 points decrease from baseline, between the complication and no-complication groups (PCS-12: 17.6% vs. 15.2%, *p* = 0.55; MCS-12: 25.2% vs. 22.4%, *p* = 0.53; Table 5).

**Table 4:** Baseline and 30-d or 1-y SF-12 scores

Baseline and 30-d SF-12 scores∗ (n = 678)	PCS-12				MCS-12			
	Baseline	*p*†	30-d	*p*†	Baseline	*p*†	30-d	*p*†
Patients without complications, n = 519	33.59 ± 11.34	-	41.94 ± 12.57	-	43.50 ± 14.98	-	49.06 ± 13.99	-
Patients with any complications, n = 159	32.23 ± 11.82	0.20	39.63 ± 13.51	0.06	40.23 ± 15.12	0.02	45.62 ± 15.24	0.01
Nonfatal stroke, n = 16	29.82 ± 11.69	0.22	39.53 ± 12.74	0.47	40.99 ± 15.74	0.54	46.99 ± 15.43	0.60
Disabling stroke, n = 6	29.14 ± 16.81	0.55	34.23 ± 12.06	0.18	35.99 ± 17.07	0.33	38.63 ± 16.27	0.18
Nondisabling stroke, n = 10	30.23 ± 8.38	0.24	42.72 ± 12.65	0.85	44.00 ± 14.97	0.92	52.01 ± 13.24	0.50
Bleeding type 3, n = 9	37.64 ± 11.01	0.30	37.52 ± 10.98	0.26	40.98 ± 17.22	0.67	41.31 ± 13.54	0.13
Major vascular complications, n = 10	36.00 ± 4.99	0.17	44.36 ± 10.03	0.47	38.68 ± 11.26	0.21	49.74 ± 11.57	0.86
AKI stage 2-4, n = 7	29.49 ± 6.34	0.14	35.21 ± 13.20	0.23	38.46 ± 6.41	0.09	44.93 ± 11.67	0.39
≧moderate PVR, n = 24	35.78 ± 13.53	0.44	42.82 ± 10.93	0.71	44.50 ± 13.99	0.74	49.72 ± 13.77	0.82
New permanent pacemaker, n = 109	31.82 ± 11.84	0.16	39.38 ± 14.11	0.08	39.64 ± 15.36	0.02	45.15 ± 15.54	0.02
Surgery or intervention, n = 6	36.52 ± 4.75	0.20	45.18 ± 8.84	0.41	42.94 ± 9.71	0.89	54.19 ± 4.94	0.05

Baseline and 1-y SF-12 scores (n = 662)‡	PCS-12				MCS-12			
	Baseline	*p*†	1-y	*p*†	Baseline	*p*†	1-y	*p*†
Patients without complications, n = 511	33.66 ± 11.34	-	40.50 ± 13.35	-	43.44 ± 14.91	-	48.15 ± 15.50	-
Patients with any complications, n = 151	32.09 ± 11.78	0.15	38.76 ± 12.96	0.15	40.51 ± 15.37	0.04	47.13 ± 16.20	0.50
Nonfatal stroke, n = 18	30.43 ± 11.14	0.24	36.76 ± 11.20	0.18	41.00 ± 15.05	0.51	49.42 ± 14.60	0.72
Disabling stroke, n = 7	29.73 ± 15.42	0.53	31.66 ± 9.97	0.06	37.88 ± 16.36	0.41	46.51 ± 16.62	0.80
Nondisabling stroke, n = 11	30.88 ± 8.23	0.30	40.01 ± 11.12	0.89	42.99 ± 14.59	0.92	51.27 ± 13.68	0.47
Bleeding type 3, n = 9	37.64 ± 11.01	0.31	41.13 ± 11.59	0.88	40.98 ± 17.22	0.68	43.86 ± 17.82	0.49
Major vascular complications, n = 9	36.80 ± 4.56	0.08	45.29 ± 6.57	0.06	40.14 ± 10.89	0.40	45.45 ± 10.55	0.47
AKI stage 2-4, n = 5	30.56 ± 7.40	0.41	37.30 ± 7.39	0.39	39.95 ± 6.80	0.32	55.78 ± 8.41	0.11
≧moderate PVR, n = 20	35.63 ± 13.82	0.54	40.09 ± 12.67	0.89	44.88 ± 13.72	0.65	49.89 ± 15.53	0.63
New permanent pacemaker, n = 102	31.42 ± 11.88	0.08	38.19 ± 13.49	0.11	39.62 ± 15.84	0.03	46.35 ± 17.00	0.33
Surgery or intervention, n = 7	35.37 ± 5.29	0.43	37.62 ± 11.06	0.52	44.46 ± 9.73	0.79	42.36 ± 8.88	0.14

Abbreviations: AKI, acute kidney injury; MCS, mental component summary; PCS, physical component summary; PVR, prosthetic valvular regurgitation; SF-12, Short Form-12.

∗ PCS and MCS scores at baseline and 30 days in 678 patients with both baseline and 30-days SF-12 data available. No imputation for missing SF-12 data was performed.

† *p* values were calculated using chi-square or Fisher exact test between the patients without early nonfatal complications and those with complications.

‡ PCS and MCS scores at baseline and 30 days in 662 patients with both baseline and 1-year SF-12 data available. Imputation for missing SF-12 data was performed in 40 patients.

**Table 5:** Changes in SF-12 from baseline to 30 d

	ΔPCS-12 (30 d - baseline)	*p*∗	ΔMCS-12 (30 d - baseline)	*p*∗
Patients without complications, n = 519	8.36 ± 11.70	-	5.56 ± 12.57	-
Patients with any complications, n = 159	7.40 ± 11.88	0.37	5.39 ± 12.67	0.89
Nonfatal stroke, n = 16	9.71 ± 12.26	0.67	6.00 ± 9.42	0.86
Disabling stroke, n = 6	5.09 ± 12.27	0.54	2.64 ± 5.47	0.26
Nondisabling stroke, n = 10	12.49 ± 12.00	0.31	8.01 ± 10.92	0.50
Bleeding type 3, n = 9	−0.13 ± 8.28	0.02	0.33 ± 16.79	0.38
Major vascular complications, n = 10	8.36 ± 10.45	1.00	11.06 ± 10.37	0.13
AKI stage 2-4, n = 7	5.73 ± 8.84	0.47	6.47 ± 8.54	0.79
Moderate PVR, n = 24	7.04 ± 12.03	0.60	5.22 ± 14.09	0.91
New permanent pacemaker, n = 109	7.57 ± 12.14	0.53	5.51 ± 12.83	0.98
Surgery or intervention, n = 6	8.66 ± 12.05	0.95	11.25 ± 9.56	0.21

	≧2.5 points decrease in PCS-12 at 30 d, n (%)	*p*∗	≧2.5 points decrease in MCS-12 at 30 d, n (%)	*p*∗

Patients without complications, n = 519	79 (15.2%)	-	116 (22.4%)	-
Patients with any complications, n = 159	28 (17.6%)	0.55	40 (25.2%)	0.53
Nonfatal stroke, n = 16	3 (18.8%)	0.72	3 (18.8%)	1.00
Disabling stroke, n = 6	2 (33.3%)	0.23	1 (16.7%)	1.00
Nondisabling stroke, n = 10	1 (10.0%)	1.00	2 (20.0%)	1.00
Bleeding type 3, n = 9	3 (33.3%)	0.15	3 (33.3%)	0.43
Major vascular complications, n = 10	1 (10.0%)	1.00	1 (10.0%)	0.70
AKI stage 2-4, n = 7	1 (14.3%)	1.00	0 (0.0%)	0.36
Moderate PVR, n = 24	6 (25.0%)	0.24	8 (33.3%)	0.32
New permanent pacemaker, n = 109	18 (16.5%)	0.85	28 (25.7%)	0.53
Surgery or intervention, n = 6	1 (16.7%)	1.00	1 (16.7%)	1.00

Changes in SF-12 from baseline to 30 days were assessed in 678 patients with both baseline and 30-day SF-12 data available. No imputation was performed for missing SF-12 data.

Abbreviations: AKI, acute kidney injury; MCS, mental component summary; PCS, physical component summary; PVR, prosthetic valvular regurgitation; SF-12, Short Form-12.

∗ *p* values were calculated between the patients without early nonfatal complications and those with complications.

There were no significant differences in the changes in PCS-12 or MCS-12 between baseline and 30-days, in the complication versus the noncomplication group (ΔPCS-12 at 30 days: 7.40 ± 11.88 vs. 8.36 ± 11.70, *p* = 0.37; ΔMCS-12 at 30 days: 5.39 ± 12.67 vs. 5.56 ± 12.57, *p* = 0.89 Table 5). When analyzed by complication type, patients with type 3 bleeding showed a significantly smaller improvement (i.e., a greater decline) in PCS-12 compared with the no-complication group (ΔPCS at 30 days: −0.13 ± 8.28, *p* = 0.02), whereas no significant differences were observed for other complication types.

### QOL at 1 Year

Baseline and 1-year SF-12 assessments were available for 662 patients, including 40 patients with imputed 1-year SF-12 scores who were alive and had not withdrawn consent at 1 year (Figure 1). At 1 year, there were no significant differences in PCS-12 and MCS-12 scores (PCS-12: 38.76 ± 12.96 vs. 40.50 ± 13.35, *p* = 0.15; MCS-12 at 1 year: 47.13 ± 16.20 vs. 48.15 ± 15.50, *p* = 0.50; Table 4), nor was there a significant difference in the incidence of QOL deterioration (≥2.5 points decrease from baseline) between the complication and no-complication groups (PCS-12: 17.9% vs. 21.9%, *p* = 0.34; MCS-12: 25.2% vs. 29.4%, *p* = 0.37; Table 6). There were no significant differences in changes from baseline to 1 year in either PCS-12 or MCS-12 between the complication and no-complication groups (ΔPCS-12 at 1 year: 6.67 ± 13.08 vs. 6.85 ± 13.47, *p* = 0.88; ΔMCS-12 at 1 year: 6.62 ± 15.14 vs. 4.71 ± 16.02, *p* = 0.18; Table 6). No significant differences were observed when each complication type was compared with the no-complication group. Sensitivity analysis excluding patients with imputed 1-year SF-12 scores (n = 622) showed similar results, with no significant differences between the complication and noncomplication groups (Supplementary Tables 2 and 3).

**Table 6:** Changes in SF-12 from baseline to 1 y

	ΔPCS-12 (1 y - baseline)	*p*∗	ΔMCS-12 (1 y - baseline)	*p*∗
Patients without complications, n = 511	6.85 ± 13.47	-	4.71 ± 16.02	-
Patients with any complications, n = 151	6.67 ± 13.08	0.88	6.62 ± 15.14	0.18
Nonfatal stroke, n = 18	6.33 ± 12.49	0.87	8.42 ± 8.92	0.11
Disabling stroke, n = 7	1.93 ± 14.34	0.40	8.63 ± 6.79	0.18
Nondisabling stroke, n = 11	9.14 ± 10.94	0.51	8.28 ± 10.37	0.29
Bleeding type 3, n = 9	3.49 ± 5.37	0.11	2.88 ± 9.58	0.59
Major vascular complications, n = 9	8.49 ± 7.08	0.52	5.30 ± 8.37	0.84
AKI stage 2-4, n = 5	6.74 ± 5.92	0.97	15.83 ± 12.46	0.12
Moderate PVR, n = 20	4.46 ± 18.84	0.58	5.00 ± 20.63	0.95
New permanent pacemaker, n = 102	6.76 ± 13.27	0.95	6.73 ± 16.37	0.26
Surgery or intervention, n = 7	2.25 ± 9.59	0.26	-2.09 ± 11.02	0.16

	≧2.5 points decrease in PCS-12 at 1 y, n (%)	*p*∗	≧2.5 points decrease in MCS-12 at 1 y, n (%)	*p*∗

Patients without complications, n = 511	112 (21.9)	-	150 (29.4)	-
Patients with any complications, n = 151	27 (17.9)	0.34	38 (25.2)	0.37
Nonfatal stroke, n = 18	5 (27.8)	0.57	2 (11.1)	0.16
Disabling stroke, n = 7	3 (42.9)	0.19	0 (0.0)	0.20
Nondisabling stroke, n = 11	2 (18.2)	1.00	2 (18.2)	0.52
Bleeding type 3, n = 9	2 (22.2)	1.00	3 (33.3)	0.73
Major vascular complications, n = 9	1 (11.1)	0.69	2 (22.2)	1.00
AKI stage 2-4, n = 5	0 (0.0)	0.59	0 (0.0)	0.33
Moderate PVR, n = 20	6 (30.0)	0.41	5 (25.0)	0.87
New permanent pacemaker, n = 102	15 (14.7)	0.13	28 (27.5)	0.79
Surgery or intervention, n = 7	3 (42.9)	0.19	3 (42.9)	0.43

Changes in SF-12 from baseline to 1 year were assessed in 662 patients with both baseline and 1-year SF-12 data available. Imputation for missing SF-12 data at 1 year was performed in 40 patients.

Abbreviations: AKI, acute kidney injury; MCS, mental component summary; PCS, physical component summary; PVR, prosthetic valvular regurgitation; SF-12, Short Form-12.

∗ *p* values were calculated between the patients without early nonfatal complications and those with complications.

## Discussion

The key findings of this post-hoc substudy of the LANDMARK trial are the following.

1. Patients with early nonfatal complications had significantly higher mortality between 30 and 365 days after TAVI compared with those without.
2. Early nonfatal complications were independently associated with 1-year mortality.
3. There were no significant differences in QOL outcomes at 1 year between patients with and without early nonfatal complications, provided patients survived.

A key novelty of this study is the evaluation of a composite of early nonfatal complications, defined according to the VARC-3 early safety endpoint, in the context of contemporary TAVI. The current study demonstrated that a composite of early nonfatal complications was associated with increased mortality, procedure- or valve-related hospitalizations, and clinical efficacy composite endpoints at 1 year. Multivariable analysis confirmed the prognostic impact of the VARC-3 early safety endpoint.

Previous studies have reported an association between individual periprocedural complications after TAVI and subsequent mortality. Arnold et al.^13^ evaluated complications occurring within 30 days after TAVI, among 3763 patients enrolled in the PARTNER 2 studies who were alive at 30 days, and reported markedly higher 1-year mortality in patients with major versus minor stroke (49.3% vs. 3.8%); major stroke was independently associated with 1-year mortality, but minor stroke was not. Avvedimento et al. investigated the impact of postprocedural neurologic events in 2924 TAVI patients and reported significantly higher 1-year mortality among patients with neurologic events compared with those without (18.2% vs. 6.3%; HR, 1.91; 95% CI, 1.23–2.97; *p* = 0.004), with early divergence of the Kaplan–Meier curves within the first 30 days after TAVI.^7^ Previous studies also reported that patients with bleeding, major vascular complications, AKI stages 2-4, and moderate PVR had higher 1-year mortality compared with those without.^6^^,^^8^, ^9^, ^10^^,^^13^^,^^14^ In contrast, the impact of new PPIs on mortality after TAVI remains controversial, with some studies suggesting no adverse effect or even a protective effect on mortality,^15^^,^^29^^,^^30^ whereas others have reported an association between PPIs and increased long-term mortality.^11^^,^^31^ In addition, not only PPI itself but also the ventricular pacing burden may influence long-term outcomes.^32^ These findings pose a question of whether the inclusion of PPI in the early safety composite endpoint is essential.

In the current study, the occurrence of composite early safety complications was associated with higher 1-year mortality. This is consistent with the findings of Gonzálvez-García et al.^12^, who reported that patients with early safety complications had a threefold higher risk of 1-year mortality, and that this association persisted in a 30-day landmark analysis. However, the relationship between individual complications and 1-year mortality was inconsistent. Major vascular complications, AKI stages 2-4, and ≥moderate PVR were associated with higher 1-year mortality, whereas stroke, type 3 bleeding, and new PPI were not. This variation may be attributed to the relatively small sample size and the lower incidence of early complications in the LANDMARK trial compared with previous TAVI studies. For example, in a substudy of the PARTNER 2 studies, the early event rates for major stroke, life-threatening bleeding, major bleeding, vascular complication, and ≥moderate paravalvular leak were 1.5, 6.9, 16.8, 6.5, and 8.1%, respectively.^13^ In contrast, in the LANDMARK trial, the rates of disabling stroke, VARC-3 type 3 bleeding, major vascular complication, and ≥moderate PVR were 0.9%, 1.3%, 1.6%, and 3.6%, respectively. In addition, patient characteristics differed substantially between studies. Compared with our cohort, prior studies included patients with higher STS-PROM scores and enrolled patients who underwent nontransfemoral TAVI. In contrast, the LANDMARK trial population predominantly consisted of low-risk patients (median STS-PROM 2.6%) treated via the transfemoral approach, which is likely associated with lower subsequent mortality, even in the presence of early complications.

Major vascular complications, AKI stages 2-4, and ≥moderate PVR were associated with increased 1-year mortality in the present study. Importantly, some of these complications are potentially preventable through meticulous procedural planning and optimization. For vascular complications, computed tomography–based preprocedural planning, ultrasound-guided femoral puncture, and appropriate use of closure device may reduce risk.^33^, ^34^, ^35^ Prevention of AKI may involve adequate periprocedural hydration, minimization of contrast volume, and the avoidance of bleeding and hemodynamic instability.^36^^,^^37^ The risk of ≥moderate PVR may be mitigated by computed tomography–based sizing, appropriate implantation depth, and the use of postdilatation when necessary.^38^ The findings of this study highlight the importance of procedural refinement and standardized techniques to improve clinical outcomes in contemporary TAVI.

The VARC-3 recommends assessing patients’ QOL at follow-up, emphasizing the importance of evaluating not only hard clinical endpoints but also patients’ perspectives.^21^ Although the Kansas City Cardiomyopathy Questionnaire (KCCQ), a disease-specific patient-reported outcome measure, is the first-line recommended tool in the VARC-3, the SF-12, a generic QOL assessment tool, is considered a suitable alternative. Although numerous studies have evaluated changes in QOL before and after TAVI, data on the association between complications and QOL are limited.

Arnold et al.^13^ assessed the relationship between early nonfatal complications and 1-year mortality (n = 3763) and QOL using KCCQ (n = 2875) and SF-12 (n = 2147) in patients undergoing TAVI from the PARTNER 2 studies. Patients without complications had an average increase of 5.0 ± 10.0 points in PCS and 3.8 ± 10.5 points in MCS at 1 year. Patients with major stroke had an average decrease in both PCS (−1.7 ± 8.8) and MCS (−0.7 ± 18.4), and patients with AKI stage 3 had an average decrease in PCS (−3.6 ± 16.6). In their multivariable analysis, major stroke, life-threatening bleeding, major bleeding, and AKI were associated with a significant reduction in PCS, whereas moderate or severe PVR was associated with a significant reduction in MCS at 1 year. Van Nuland et al.^14^^,^^15^ assessed the impact of stroke, bleeding and PPI after TAVI on QOL during the first year after TAVI, using data from the POPular TAVI trial. In univariable analysis, there were no significant differences in PCS and MCS between patients with and without major bleeding, whereas patients with stroke had a lower PCS at 3 months and a lower MCS at 3 and 12 months.^14^ No significant differences in QOL were observed between patients with and without new PPI after TAVI.^15^ A substudy of the SCOPE I trial also found no significant difference in QOL outcomes, as measured by the KCCQ, between those with and without PPI.^30^ In contrast, a report from the FRANCE-TAVI registry identified new PPI as a predictor of deterioration in QOL, as assessed by the EuroQoL-5-dimension-5-level, at 1 year.^39^

The QOL data from the current analysis showed that patients who survived early complications eventually recovered their QOL by 1 year. The MCS-12 in the complication group was lower than that in the no-complication group at baseline and at 30 days but was similar by 1 year. Indeed, the increase in MCS score from baseline to 1 year tended to be higher in the complication group (6.62 ± 15.14 vs. 4.71 ± 16.02, *p* = 0.18). In contrast, the PCS-12 score did not differ between the complication and no-complication groups throughout the 1-year follow-up. These results are at variance with previous studies, such as PARTNER 2, which showed a significant negative impact of complications on 1-year QOL.^13^ Reassuringly, in the contemporary era, early post-TAVI complications have minimal impact on QOL at 1-year, provided that patients survive to 1 year.

### Limitations

There are several limitations in the current study. First, this was a post-hoc substudy of the LANDMARK trial and the analyses were not prespecified; therefore, the findings should be interpreted as hypothesis-generating. Multiple comparisons involving numerous *p* values may have increased the risk of type I error. Second, the number of patients with each specific complication was relatively small, which may have limited the statistical power to detect differences in outcomes across individual complication subgroups. Third, health-related QOL was assessed using the generic SF-12 questionnaire rather than disease-specific instruments such as the KCCQ or the Toronto Aortic Stenosis Quality of Life Questionnaire. Notably, the KCCQ is recommended as a first-line measure by the VARC-3. The absence of KCCQ data reduces comparability with other studies and may have limited the sensitivity to detect clinically meaningful changes in patient-reported outcomes. Fourth, the analysis was restricted to patients who survived the first 30 days, which may have introduced survivorship bias. Fifth, although VARC-3 includes type 2 bleeding in the early safety endpoint, it was not included in the LANDMARK trial. Sixth, the study population predominantly consisted of low-risk patients, and almost all procedures were performed via the transfemoral approach. Therefore, the generalizability of the present findings to higher-risk populations or to patients undergoing TAVI via alternative access routes may be limited.

## Conclusions

Early nonfatal complications after TAVI, as defined according to the VARC-3 early safety endpoint, have a significant prognostic impact at 1-year follow-up. However, once patients survive these early complications, their QOL recovers to the same level as that of patients without complications at 1 year.

## Funding

The LANDMARK trial is funded by Meril Life Sciences.

## Ethics Statement

The study was approved by the ethics committees of all participating centers and conducted in accordance with Good Clinical Practice guidelines and the Declaration of Helsinki.

## Disclosure Statement

Niels van Royen reports grant funding and personal fees from Abbott; grants from Philips, Biotronik, and Medtronic; speaker fees from MicroPort, Bayer, and RainMed Medical outside the submitted work; and travel support to attend meeting from Meril Life Sciences. Ignacio J. Amat-Santos reports being a proctor for Abbot, Boston Scientific, Medtronic, Meril Life Sciences, and MicroPort. Matjaz Bunc reports being a proctor of Abbott, Medtronic, Meril Life Sciences, and Edwards Lifesciences. Daniel Unic reports speaker honoraria from Medtronic and Meril Life Sciences; being a proctor of Medtronic, Abbott, and Meril Life Sciences; and is a member of the Medtronic surgical advisory board. Bela Merkely reports institutional grants from Biotronik, Boehringer Ingelheim, Daiichi Sankyo, Duke Clinical Institute, Eli Lilly, and Novartis; and speaker fees from Boehringer Ingelheim, Daiichi Sankyo, Duke Clinical Institute, and Novartis. Renicus S. Hermanides reports speaker fees from Amgen, Edwards Lifesciences, and Meril Life Sciences outside the submitted work. Marcin Protasiewicz reports speaker fees from Meril Life Sciences, Boston Scientific, and Medtronic. Pedro L. Martin reports a proctorship grant from Meril Life Sciences and an educational event grant from Medtronic. Axel Linke received grants from Edward Lifesciences and Novartis; received speaker honoraria from Edwards Lifesciences, Abiomed, Abbott, Boston Scientific, Novartis, Pfizer, BMS, Daiichi Sankyo, AstraZeneca, Boehringer Ingelheim, Meril Life Sciences, and Corvia; received travel support from AstraZeneca, Abbott, Meril Life Sciences, Abiomed, and Boston Scientific; is a partial patent holder with Boston Scientific; and is a stock option holder with Pi-Cardia, Transverse Medical, and Filterlex. Alfonso Ielasi reports speaker fees and being a proctor of Medtronic, Meril Life Sciences, and SMT. Konstantinos Toutouzas reports proctorship with Abbott, Meril Life Sciences, and Medtronic; reports consulting fees from Gore Medical; and is a Board member of the Hellenic Society of Cardiology. Manuel de Sousa Almeida reports consulting fees from Medtronic. Matteo Montorfano reports consulting fees and speaker fees from Medtronic, which were paid to the author’s institution. Francesco Bedogni reports consulting fees and being a proctor of Medtronic, Meril, Abbott, and Edwards Lifesciences. Manuel Pan reports lecture fees from Medtronic and Abbott. Oskar Angeras reports institutional research grant from Abbott and Medtronic; and proctorship and speaker fees with Meril Life Sciences, Medtronic, and Abbott. Won-Keun Kim reports honoraria or consultancy fees from Edwards Lifesciences; consulting fees from Boston Scientific, Meril Life Sciences, and Abbott; and participation on data and safety monitoring board for HID Imaging and P&F. Jürgen Rothe reports personal fees for consulting/proctoring from Meril Life Sciences, Medtronic, and Qatna; and travel support for attending meetings from Meril Life Sciences, Edwards Lifesciences, Abbott, Medtronic, and Boston Scientific. Scot Garg reports speaker fees from Novartis and consultancy fees from Biosensors. Ashokkumar Thakkar and Udita Chandra are full employees of Meril Life Sciences. Pieter C. Smits reports that he received institutional grants from Abbott Vascular and Sahajanand Medical Technologies; received consultant fees/honoraria from Abbott Vascular, Elixir, Microport, SMT, and Terumo; and is a shareholder of CERC. Marie-Claude Morice reports that she is shareholder and CEO of CERC, a CRO involved in the trial; and is a minor shareholder of Electroducer. Andreas Baumbach reports consultation fees from Faraday and Meril Life Sciences and serving on the data safety monitoring board or advisory board for Cordis. Patick W. Serruys reports consultancy for SMT, Novartis, and Meril Life Sciences.

The other authors had no conflicts to declare.
