# Supplementary material for: Impact of Early Nonfatal Complications After Transcatheter Aortic Valve Implantation on 1-Year Mortality and Quality of Life: Insights From the LANDMARK Trial
Source: Struct Heart. 2026 Jul 24;10(9):101101. doi: 10.1016/j.shj.2026.101101 (PMC13545709; doi:10.1016/j.shj.2026.101101)
Supplement: Supplementary file 1 [file mmc1.docx]

**Supplementary materials**

**Appendix. Method of multiple imputation for missing SF-12 at 1 year**

Missing data for the SF-12 was imputed using Multiple Imputation by Chained Equations (e.g. MICE). This method assumes that data are Missing at Random (MAR) and iteratively imputes missing values based on observed data. SF-12 data was imputed as a continuous outcome and then converted into a dichotomous variable to assess a decrease of ≥2.5 points in SF-12 scores. Imputation was performed only for missing SF-12 at 1 year in patients who were alive and had not withdrawn their consent.

The following parameters were used for multiple imputation for missing SF-12: age, gender, BMI, STS-PROM score, hypertension, COPD, diabetes, previous stroke, coronary artery disease, peripheral vascular disease, previous MI, previous PCI, previous CABG, bicuspid valve, mitral insufficiency, aortic annulus area, mean pressure gradient, stroke volume, LVEF, PCS/MCS at preprocedural baseline, PCS/MCS at 30 days, 6 minute-walk test at preprocedural baseline, 6 minute-walk test at 30 days, NYHA at preprocedural baseline.

**Supplementary Table 1. Other clinical outcomes from 30 to 365 days after TAVI**

| **From 30-day FU to 1-year FU** | **With early non-fatal complications,  n=177** | **Without early non-fatal complications,  n=565** | **p- values** |
| --- | --- | --- | --- |
| All cause death | 14 (8.0%) | 22 (3.9%) | 0.03 |
| All stroke | 4 (2.4%) | 9 (1.6%) | 0.52 |
| Fatal stroke | 1 (0.6%) | 2 (0.4%) | 0.68 |
| Disabling stroke | 1 (0.6%) | 5 (0.9%) | 0.70 |
| Non-disabling stroke | 2 (1.2%) | 2 (0.4%) | 0.21 |
| Procedure/valve-Hospitalization | 10 (5.8%) | 6 (1.1%) | <0.01 |
| CV hospitalization | 18 (10.6%) | 45 (8.1%) | 0.32 |
| All hospitalization | 22 (12.9%) | 54 (9.7%) | 0.23 |
| Clinical efficacy composite endpoint (all-cause death, all stroke and procedure/valve-related hospitalization) | 22 (12.5%) | 33 (5.9%) | <0.01 |
| Decrease of SF-12≧2.5 points in Both PCS-12 and MCS-12 (with imputation at 1 year) | 11 (7.3%)  (n=151) | 56 (n=11.0%) (n=511) | 0.25 |
| Decrease of ≧2.5 points PCS-12 AND/OR MCS-12 (with imputation at 1 year) | 54 (35.8%) (n=151) | 206 (40.3%) (n=511) | 0.36 |
| Extended clinical efficacy endpoint (all-cause death, all stroke, procedure/valve-related hospitalization and ≧2.5 points decrease in Both PCS and MCS) | 32 (18.1%)  (n=177) | 86 (15.2%) (n=565) | 0.43 |
| Extended clinical efficacy endpoint (all-cause death, all stroke, procedure/valve-related hospitalization and ≧2.5 points decrease in PCS OR MCS) | 73(41.2%)  (n=177) | 233 (41.2%) (n=565) | 1.00 |

MCS=mental component summary, PCS=physical component summary, TAVI=transcatheter aortic valve implantation

**Supplementary Table 2. Baseline and 1-year SF-12 scores in patients with both baseline and 1-year SF-12 available without imputation**

|  | **PCS-12** | | | | **MCS-12** | | | |
| --- | --- | --- | --- | --- | --- | --- | --- | --- |
|  | **Baseline** | **P*** | **1-year** | **P*** | **Baseline** | **P*** | **1-year** | **P*** |
| Patients **without** complications, n=481 | 33.67 ± 11.31 | - | 40.54 ± 13.58 | - | 43.33 ± 14.90 | - | 48.10 ± 15.79 | **-** |
| Patients **with** any complications, n=141 | 32.70 ± 11.71 | 0.39 | 38.94 ± 12.99 | 0.21 | 40.64 ± 15.24 | 0.07 | 47.40 ± 16.22 | 0.65 |
| Non-Fatal stroke, n=17 | 30.65 ± 11.45 | 0.3 | 36.16 ± 11.24 | 0.14 | 40.54 ± 15.38 | 0.47 | 49.35 ± 15.05 | 0.74 |
| Disabling stroke, n=6 | 30.23 ± 16.83 | 0.64 | 29.10 ± 8.01 | 0.02 | 36.05 ± 17.13 | 0.35 | 45.83 ± 18.10 | 0.77 |
| Non-disabling stroke, n=11 | 30.88 ± 8.23 | 0.29 | 40.01 ± 11.12 | 0.88 | 42.99 ± 14.59 | 0.94 | 51.27 ± 13.68 | 0.47 |
| Bleeding type 3, n=9 | 37.64 ± 11.01 | 0.31 | 41.13 ± 11.59 | 0.88 | 40.98 ± 17.22 | 0.7 | 43.86 ± 17.82 | 0.50 |
| Major vascular complications, n=9 | 36.80 ± 4.56 | 0.08 | 45.29 ± 6.57 | 0.07 | 40.14 ± 10.89 | 0.41 | 45.45 ± 10.55 | 0.48 |
| AKI stage 2-4, n=5 | 30.56 ± 7.40 | 0.40 | 37.30 ± 7.39 | 0.39 | 39.95 ± 6.80 | 0.33 | 55.78 ± 8.41 | 0.11 |
| ≧moderate PVR, n=19 | 36.46 ± 13.68 | 0.39 | 40.23 ± 13.00 | 0.92 | 45.56 ± 13.75 | 0.5 | 49.69 ± 15.93 | 0.67 |
| New permanent pacemaker, n=94 | 32.10 ± 11.77 | 0.23 | 38.47 ± 13.48 | 0.18 | 39.75 ± 15.59 | 0.04 | 46.80 ± 16.97 | 0.49 |
| Surgery or intervention, n= 7 | 35.37 ± 5.29 | 0.44 | 37.62 ± 11.06 | 0.52 | 44.46 ± 9.73 | 0.77 | 42.36 ± 8.88 | 0.14 |

PCS and MCS scores at baseline and 1 year in 622 patients with both baseline and 30-day SF-12 data available without imputation for missing SF-12 data.

*P values were calculated using Chi square or Fisher’s exact test between the patients without early non-fatal complications and those with complications.

AKI=acute kidney injury, PVR=prosthetic valvular regurgitation, TAVI=transcatheter aortic valve implantation

**Supplementary Table 3. Changes in SF-12 from baseline to 1 year without imputation**

|  | **ΔPCS-12**  **(1 year - baseline)** | **P*** | **ΔMCS-12**  **(1 year - baseline)** | **P*** |
| --- | --- | --- | --- | --- |
| Patients **without** complications, n=481 | 6.86 ± 13.48 | **-** | 4.77 ± 16.15 | **-** |
| Patients **with** any complications, n=141 | 6.24 ± 13.16 | 0.62 | 6.76 ± 15.25 | 0.18 |
| Non-Fatal stroke, n=17 | 5.51 ± 12.36 | 0.66 | 8.8 ± 9.04 | 0.10 |
| Disabling stroke, n=6 | -1.13 ± 12.96 | 0.19 | 9.77 ± 6.67 | 0.13 |
| Non-disabling stroke, n=11 | 9.14 ± 10.94 | 0.51 | 8.28 ± 10.37 | 0.30 |
| Bleeding type 3, n=9 | 3.49 ± 5.37 | 0.10 | 2.88 ± 9.58 | 0.58 |
| Major vascular complications, n=9 | 8.49 ± 7.08 | 0.52 | 5.3 ± 8.37 | 0.86 |
| AKI stage 2-4, n=5 | 6.74 ± 5.92 | 0.97 | 15.83 ± 12.46 | 0.12 |
| ≧moderate PVR, n=19 | 3.78 ± 19.1 | 0.49 | 4.13 ± 20.82 | 0.9 |
| New permanent pacemaker, n=94 | 6.38 ± 13.39 | 0.75 | 7.05 ± 16.55 | 0.22 |
| Surgery or intervention, n=7 | 2.25 ± 9.59 | 0.25 | -2.09 ± 11.02 | 0.15 |
|  | | | | |
|  | **≧2.5 points decrease in PCS-12 at 1 year, n (%)** | **P*** | **≧2.5 points decrease in MCS-12 at 1 year, n (%)** | **P*** |
| Patients **without** complications, n=481 | 107 (22.2%) | - | 139 (29.0%) | - |
| Patients **with** any complications, n=141 | 27 (19.2%) | 0.50 | 36 (25.5%) | 0.50 |
| Non-Fatal stroke, n=17 | 5 (29.4%) | 0.55 | 2 (11.8%) | 0.17 |
| Disabling stroke, n=6 | 3 (50.0%) | 0.13 | 0 (0.0%) | 0.19 |
| Non-disabling stroke, n=11 | 2 (18.2%) | 1.00 | 2 (18.2%) | 0.74 |
| Bleeding type 3, n=9 | 2 (22.2%) | 1.00 | 3 (33.3%) | 0.72 |
| Major vascular complications, n=9 | 1 (11.1%) | 0.69 | 2 (22.2%) | 1.00 |
| AKI stage 2-4, n=5 | 0 (0.0%) | 0.59 | 0 (0.0%) | 0.33 |
| ≧moderate PVR, n=19 | 6 (31.6%) | 0.40 | 5 (26.3%) | 1.00 |
| New permanent pacemaker, n=94 | 15 (16.0%) | 0.22 | 26 (27.7%) | 0.91 |
| Surgery or intervention, n=7 | 3 (42.7%) | 0.19 | 3 (42.7%) | 0.42 |

Changes in SF-12 from baseline to 1 year were assessed in 622 patients with both baseline and 1-year SF-12 data available without imputation for missing SF-12 data at 1 year.

*P values were calculated between the patients without early non-fatal complications and those with complications.

AKI=acute kidney injury, PVR=prosthetic valvular regurgitation, TAVI=transcatheter aortic valve implantation

**Supplementary Figure 1**. Hazard ratios and forest plot of each complication for 1-year mortality

The non-complication group was used as a reference.

**Supplementary Figure 2**. All-cause mortality according to the number of early non-fatal complications

| Total number of early non-fatal complications | Number of patients | All-cause mortality at 1 year,  N (KM%) |
| --- | --- | --- |
| 0 | 565 | 22 (3.9%) |
| 1 | 136 | 9 ( 6.7%) |
| 2 | 33 | 5 ( 15.4%) |
| 3 | 7 | 0 (0.0%) |
| 4 | 1 | 0 (0.0%) |
